# ‘Carriers of V‐LH among 1593 Baltic men have significantly higher serum LH’

**DOI:** 10.1111/andr.12022

**Published:** 2015-03-26

**Authors:** A. M. Punab, M. Grigorova, M. Punab, M. Adler, T. Kuura, O. Poolamets, V. Vihljajev, B. Žilaitienė, J. Erenpreiss, V. Matulevičius, M. Laan

**Affiliations:** ^1^ Human Molecular Genetics Research GroupInstitute of Molecular and Cell BiologyUniversity of TartuTartuEstonia; ^2^ Andrology UnitTartu University ClinicsTartuEstonia; ^3^ Lithuanian University of Health SciencesMedical AcademyInstitute of EndocrinologyKaunasLithuania; ^4^ Andrology LaboratoryRiga Stradins UniversityRigaLatvia

**Keywords:** *LHB* gene, luteinizing hormone, testes volume, V‐LH

## Abstract

Luteinizing hormone (LH) is a pituitary heterodimeric glycoprotein essential in male and female reproduction. Its functional polymorphic variant (V‐LH) is determined by two missense mutations (rs1800447, A/G, Trp8Arg; rs34349826, A/G, Ile15Thr) in the LH β‐subunit encoding gene (*LHB*; 19q13.3; 1111 bp; 3 exons). Among women, V‐LH has been associated with higher circulating LH and reduced fertility, but the knowledge of its effect on male reproductive parameters has been inconclusive. The objective of this study was to assess the effect of V‐LH on hormonal, seminal and testicular parameters in the Baltic young men cohort (*n* = 986; age: 20.1 ± 2.1 years) and Estonian idiopathic infertility patients (*n* = 607; 35.1 ± 5.9 years). V‐LH was detected by genotyping of the underlying DNA polymorphisms using PCR‐RFLP combined with resequencing of a random subset of subjects. Genetic associations were tested using linear regression under additive model and results were combined in meta‐analysis. No significant difference was detected between young men and infertility patients for the V‐LH allele frequency (11.0 vs. 9.3%, respectively). V‐LH was associated with higher serum LH in both, the young men cohort (*p *=* *0.022, allelic effect = 0.26 IU/L) and the idiopathic infertility group (*p *=* *0.008, effect = 0.59 IU/L). In meta‐analysis, the statistical significance was enhanced (*p *=* *0.0007, resistant to Bonferroni correction for multiple testing; effect = 0.33 IU/L). The detected significant association of V‐LH with increased serum LH remained unchanged after additional adjustment for the SNPs previously demonstrated to affect LH levels (*FSHB* ‐211G/T, *FSHR* Asn680Ser, *FSHR* ‐29A/G). Additionally, a suggestive trend for association with reduced testicular volume was observed among young men, and with lower serum FSH among infertility patients. The V‐LH carrier status did not affect sperm parameters and other circulating reproductive hormones. For the first time, we show a conclusive contribution of V‐LH to the natural variance in male serum LH levels. Its downstream clinical consequences are still to be learned.

## Introduction

Luteinizing hormone (LH) is a pituitary‐derived heterodimeric glycoprotein that shares the common α‐subunit with FSH, hCG and TSH, but possesses a unique β‐subunit conferring the biological specificity of LH (Pierce & Parsons, 1981; Gharib *et al*., 1990). In both genders, LH is essential for reproduction and its action is mediated through binding to its receptors in testes and ovaries. In men, LH stimulates the production of testosterone by testicular Leydig cells. In women, LH triggers ovulation and the development of the corpus luteum to prepare the endometrium for an implantation (Moyle & Campbell, 1996).

A functional variant of LH (V‐LH), first detected in Finland (Pettersson *et al*., 1992) and Japan (Furui *et al*., 1994), is determined by two missense mutations (Trp8Arg, Ile15Thr) in the gene coding for the LH β‐subunit (*LHB*; 1111 bp; 3 exons; chr. 19q13.3) (Pettersson *et al*., 1994). The two SNPs coding for V‐LH (A‐>G, rs1800447; A‐>G, rs34349826) are located in the *LHB* exon 2 and exhibit complete linkage disequilibrium (LD) (Nilsson *et al*., 1998). V‐LH seems to be a universally common variant with the average minor allele frequency among Europeans 8% (range 7.34–11.8%; Huhtaniemi *et al*., 2010). The change from isoleucine to threonine (Ile15Thr) creates an extra glycosylation site Asn^13^‐Ala‐Thr in V‐LH compared to the wild‐type (WT) LH (four vs. three oligosaccharide chains) (Suganuma *et al*., 1996). As the half‐life of pituitary gonadotropins depends on the number and type of oligosaccharide side chains (Morell *et al*., 1971; Strott, 2002), V‐LH is predicted to have an altered half‐life in circulation. However, it is not certain whether the circulating V‐LH has increased or decreased stability compared to WT‐LH and the published data are also inconsistent concerning the half‐life of these hormone variants (Haavisto *et al*., 1995; Suganuma *et al*., 1996; Manna *et al*., 2002; Wide *et al*., 2010). In addition, the V‐LH determining polymorphisms were reported to be in LD with a certain *LHB* promoter haplotype characterized by significantly higher basal transcriptional activity (Jiang *et al*., 1999).

Although the V‐LH was reported to possess an increased in vitro bioactivity (Manna *et al*., 2002), most clinical observations suggest that V‐LH is functionally less potent than WT‐LH (Nagirnaja *et al*., 2010). Case–control association studies in male patients have suggested V‐LH as a risk factor for cryptorchidism (Kaleva *et al*., 2005) and testicular cancer (Elkins *et al*., 2003). A small‐scale study (*n* = 49) investigating the onset and progression of puberty in healthy boys reported slower progression of puberty and lower testes volume in V‐LH carriers (Raivio *et al*., 1996). In a European cohort of middle‐aged and elderly men (EMAS study: *n *= 2748 men, eight European countries), V‐LH was associated with reduced levels of serum LH (Huhtaniemi *et al*., 2010). In female patients, several studies have associated V‐LH with subfertility because of ovulatory disorders (Furui *et al*., 1994; Takahashi *et al*., 1998, 1999; Du *et al*., 2012). Among women, Du *et al*. (2012) showed an association of V‐LH with higher levels of serum LH and Rajkhowa *et al*. (1995) has reported increased levels of testosterone, oestrogen and SHBG.

Taken together, the published studies are inconsistent regarding the effect of V‐LH on serum LH levels and its downstream physiological consequences. One of the reasons might be that the majority of association studies with V‐LH have been carried out on relatively small patient groups and for limited sets of clinical parameters (Nagirnaja *et al*., 2010). In addition, most of the studies have determined V‐LH using a technically demanding immunoassay‐based approach, which measures the two alternative isoforms of LH. Only a handful of studies have used direct and robust detection of the two DNA variants underlying V‐LH (A‐>G, rs1800447, Trp8Arg; A‐>G, rs34349826, Ile15Thr) either by sequencing or genotyping (Ramanujam *et al*., 2000; Lee *et al*., 2003; Berger *et al*., 2005; Du *et al*., 2012). To overcome the limitations of previous studies, we explored for the first time the effect of V‐LH on a comprehensive set of hormonal, seminal and testicular parameters in a large and well‐characterized sample set consisting of the population‐based Baltic young men cohort (*n* = 986) and the study group of Estonian idiopathic infertility patients (*n *= 607).

## Materials and Methods

### Ethics statement

The study has been approved by the Ethics Committee of Human Research of the University Clinic of Tartu, Estonia (approval date 27.01.2003), the Ethics Committee of Riga Stradins University, Latvia (23.04.2003) and the Regional Ethics Committee of Kaunas, Lithuania (approval no. 13, 2003).

### The Baltic young male cohort

The Baltic young male cohort was recruited between May 2003 and June 2004 among the participants in a prospective study Environment and Reproductive Health (EU 5th FP project QLRT‐2001‐02911) in parallel at three study centres (Tartu, Estonia; Riga, Latvia; Kaunas, Lithuania). The study group consist of military conscripts from Estonia, Latvia and Lithuania (age: 20.1 ± 2.1 years; marital and parenthood status not documented). The recruitment and phenotyping protocols at the participating centres were identical. Study participation was voluntary and written informed consent was obtained from all subjects. Details of the study group formation were described previously (Punab *et al*., 2002). Men were recruited to the study at the Centre of Andrology, University Clinic of Tartu, Estonia (*n* = 578; all born and living in Estonia), at the Riga Family and Sexual Problems Centre, Latvia (*n *= 300; all born and living in Latvia) and at the specialized laboratory of the Institute of Endocrinology, Kaunas University of Medicine (*n* = 326; all born and living in Lithuania). In genetic association studies, cohort participants with clinical factors leading to strongly deviated reproductive physiology (lack of spermatozoa in ejaculate, i.e. azoospermia, *n *= 2; cryptorchidism, *n* = 13; abuse of anabolic steroids, *n* = 1; orchitis with unilateral testis damage, *n* = 1) or incomplete clinical data (*n* = 9), have been excluded. For the current study, DNA samples of a subset (*n* = 192) of the full cohort were unavailable for genotyping. The final number of Baltic young men analysed for the V‐LH variant was 986 (Table 1A).

**Table 1:** Characteristics of the study groups

Parameter	Baltic male cohort study group (*n *=* *986)		Estonian idiopathic infertility group (*n *= 607)	
	Mean ± SD	Median (25–75th percentile)	Mean ± SD	Median (25–75th percentile)
A. General characteristics				
Age (years)	20.1 ± 2.1	19.8 (18.5–21.5)	35.1 ± 5.9	30.7 (27.1–34.9)
BMI	22.4 ± 2.6	22.1 (20.7–23.6)	26.8 ± 4.5^a^	26.0 (23.7–29.3)
Abstinence period (hours)	107.7 ± 63.1	86.0 (63.0–134.0)	75.3 ± 44.8	72.0 (48.0–96.0)
Total testes volume (mL)	49.1 ± 10.4	50.0 (41.0–55.0)	40.2 ± 10.2	40.0 (34.0–47.0)
Sperm concentration (10^6^/mL)	80.7 ± 72.1	63.3 (34.7–105.8)	8.0 ± 5.9	7.0 (2.6–13.0)
B. Genotyping data of V‐LH (Trp8Arg, rs1800447)				
Allele frequencies, % (number of chromosomes)				
A	89.0 (1756)		90.7 (1101)	
G	11.0 (216)		9.3 (113)	
Genotype frequencies, % (number of genotype carriers)^b^				
A/A	79.1 (780)		82.4 (500)	
A/G	19.9 (196)		16.6 (101)	
G/G	1.0 (10)		1.0 (6)	
χ^2^ test			*p *=* *0.27^c^	

a Data for BMI available for 318 patients of the Estonian idiopathic infertility group.

b Hardy–Weinberg Equilibrium test in Baltic male cohort and Estonian idiopathic infertility group, *p *=* *0.55 and *p* = 0.72, respectively.

c *p*‐value from Chi‐squared test for differences in V‐LH genotype distribution between Estonian idiopathic infertility group and Baltic male cohort.

### Estonian idiopathic infertility patients

The study group of oligozoospermic Estonian men with idiopathic infertility (*n* = 750) was recruited at the Andrology Centre, Tartu University Clinics between June 2003 and August 2008 and consisted of male partners of couples failing to conceive a child for a period of ≥ 12 months. Oligozoospermia was diagnosed according to the World Health Organization (WHO) criteria valid at the time of recruitment (sperm concentration < 20 mln/mL; World Health Organization, 1999). Phenotyping protocol was identical with that in Baltic young male cohort (Punab *et al*., 2002); the details of the formation of the study group are described elsewhere (Punab, 2007). In brief, all study participants were of white European ancestry, born and living in Estonia. All men with causal factors for male factor infertility (obstruction, cryptorchidism, chromosomal abnormalities, Y chromosome deletions, hypogonadotrophic hypogonadism, testicular diseases, sexual dysfunctions, androgen abuse, severe traumas and operation in genital area, chemo‐ and radiotherapy) were excluded from the analyses resulting in a study group consisting of 688 participants. For the current project, also patients with azoospermia, that is lack of spermatozoa in ejaculate, *n* = 47) were additionally excluded from the genetic analysis. For the current study, DNA samples of a subset (*n* = 34) of the full cohort were unavailable for genotyping. The final number of analysed subjects successfully genotyped for V‐LH was 607 (age: 35.1 ± 5.9 years). Among the included infertility patients (sperm concentration, < 20 mln/mL), 376 subjects also fulfilled the latest World Health Organization, 2010 criteria for oligozoospermia (sperm count below 39 × 10^6^/ejaculate; World Health Organization, 2010).

### Hormone assays

For all participants of the study, venous blood was obtained from the cubital vein in the morning and serum was separated immediately. Serum sampling period for the Baltic cohort was from 08.00 to 13.00, and for the Estonian infertility patients from 08.00 to 11.00.

For the Baltic young men cohort, serum levels of FSH, LH and total testosterone were determined using time‐resolved immunofluorometric assays (Delfia, Wallac, Turku, Finland), estradiol by radioimmunoassay (Pantex, Santa Monica, CA, USA) and Inhibin B by a specific two‐sided enzyme immunometric assay (Serotec, Oxford, UK) at the Department of Growth and Reproduction in Copenhagen, Rigshospitalet, Denmark in the framework of the Environment and Reproductive Health (EU 5th FP project QLRT‐2001‐02911). The intra‐ and inter‐assay coefficients of variation (CV) for measurement of both FSH and LH were 3 and 4.5%, for total testosterone < 8 and < 5%, for estradiol 7.5 and 13% and for Inhibin B 15 and 18%, respectively.

For the Estonian idiopathic infertility patients, the FSH, LH, total testosterone and estradiol levels of blood serum were measured using the Immulite automated chemiluminescence immunoassay analyser (Immulite; Diagnostic Products Corp., Los Angeles, CA, USA) according to manufacturer's instructions, at the United Laboratories, University of Tartu Clinics. The intra‐ and inter‐assay CV were 4.2 and 8% for FSH; 4.0 and 7.1% for LH; 6.3 and 9.4% for testosterone; 7.5 and 13% for estradiol.

### Semen analysis and physical examination

Semen samples were obtained by masturbation and all semen values were determined in accordance with the World Health Organization (WHO) criteria valid at the time of recruitment (World Health Organization, 1999). In brief, after ejaculation, the semen was incubated at 37˚C for 30–40 min for liquefaction. Semen volume was estimated by weighing the collection tube with the semen sample and subsequently subtracting the predetermined weight of the empty tube assuming 1 g = 1 mL. For assessment of the sperm concentration, the samples were diluted in a solution of 0.6 mol/L NaHCO3 and 0.4% (v/v) formaldehyde in distilled water. The sperm concentration was assessed using the improved Neubauer haemocytometers.

Patients were examined by clinical investigators who had passed special clinical training. Physical examination for the assessment of genital pathology and testicular size was performed with the man in standing position. If necessary, pathologies were clarified further with the men in supine position. The orchidometer (made of birch wood, Pharmacia & Upjohn, Denmark) was used for the assessment of testicular size. The total testes volume is the sum of right and left testicles.

### Genotyping procedure and data

Genotyping by PCR – RFLP (restriction fragment length polymorphism) analysis was performed for screening *LHB* Trp8Arg (A‐>G, rs1800447; *Nco*I, WT‐LH specific restriction‐site) and Ile15Thr (A‐>G, rs34349826; *Bse*GI, V‐LH specific restriction‐site) polymorphisms according to the method described by Elter *et al*. (1999). All the 1593 study subjects were initially screened for the Trp8Arg polymorphism. The genotypes of the V‐LH variant carriers (both heterozygotes and homozygotes) were confirmed by *Bse*GI, cutting specifically V‐LH.

**Figure 1:**
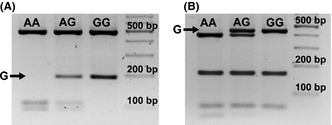
Genotyping of the V‐LH determining genetic variants Trp8Arg (A‐>G, rs1800447) and Ile15Thr (A‐>G, rs34349826) after amplification of the targeted *LHB* region (662 bp). (A) RFLP analysis with *Nco*I restriction enzyme detecting the alleles of rs1800447 (allele‐specific RFLP bands are underlined): AA‐ homozygote, fragments 473, 96 and 85; GG‐homozygote: fragments 473, and 185 bp. (B) RFLP analysis with *Bse*GI restriction enzyme detecting the alleles of rs34349826 (allele‐specific RFLP bands are underlined): AA‐homozygote, fragments 392, 165, 62 and 43 bp; GG‐homozygote: fragments 436, 165 and 62 bp. The black arrow points to the fragment characteristic to the *LHB* variant with the G‐allele (V‐LH), ‘AG’ lane indicated PCR‐RFLP pattern from a heterozygote individual. Enzyme digested PCR products were separated on 2.5% agarose gel containing EtBr.

A 662 bp fragment in the *LHB* genic region containing the V‐LH determining Trp8Arg and Ile15Thr polymorphisms was amplified using a published primer pair (forward: 5′ GAAGCAGTGTCCTTGTCCCA C 3′; reverse: 5′ GAAGAGGAGGCCTGAGAGTT 3′; Elter *et al*., 1999). The PCR product was digested either by the *Nco*I (Trp8Arg; A‐>G) or by *Bse*GI (Ile15Thr; A‐>G) restriction enzymes (Thermo Scientific, US) and the restriction fragments were separated in 2.5% agarose gel and 0.5 X Tris‐borate EDTA buffer. The *Nco*I digestion (Trp8Arg) of PCR products produced three DNA fragments (85 bp, 96 bp, 473 bp) corresponding to the A/A‐genotype in WT‐homozygotes, four fragments (85 bp, 96 bp, 185 bp, 473 bp) for the A/G‐ heterozygotes and two fragments referring to the G/G‐genotype in V‐LH homozygotes (185 bp and 473 bp; Fig.**** 1A). The *Bse*GI digestion (Ile15Thr) produced four DNA fragments (392 bp, 165 bp, 62 bp, 43 bp) in the A/A‐homozygotes corresponding to WT‐LH carriers, five fragments (436 bp, 392 bp, 165 bp, 62 bp, 43 bp) in the A/G‐heterozygotes and three fragments (436 bp, 165 bp, 62 bp) for the G/G‐genotype carriers referring to the V‐LH homozygotes (Fig. 1B). As a further inner quality control, every tenth individual was subjected to direct sequencing to blindly verify the RFLP‐based genotype detection. Sequencing analysis was performed as described by Hallast *et al*. (2005). There was no difference in the results of the three methods used for confirming V‐LH status.

### Data analysis

Mean, standard deviation, median and 25–75th percentiles were calculated for general characteristics (age, BMI, ejaculation abstinence period) and main outcome variables (hormonal and seminal variables, total testes volume) using PASW software Grad Pack 18.0 (SPSS Inc., Chicago, IL, USA).

Tests for Hardy–Weinberg equilibrium (HWE) of the genotyped SNPs and genetic association testing with male reproductive parameters were implemented in PLINK, version 1.07 (http://pngu.mgh.harvard.edu/purcell/plink/) (Purcell *et al*., 2007). The natural log‐transformation was used to obtain an approximate normal distribution of values for all studied quantitative parameters except free testosterone percentage. SNP‐trait association tests were performed using linear regression, assuming an additive genetic model. In the Baltic cohort, regression testing was adjusted for age, BMI, smoking status, recruitment centre; hormone measurements were additionally corrected for blood sampling hour, and semen parameters were corrected for ejaculation abstinence period according to the analysis settings described previously (Table S1; Grigorova *et al*., 2011). In the study group of Estonian idiopathic infertility patients, adjustment for age was applied for all parameters and abstinence period was used as an additional confounder in association tests with semen parameters. In an alternative test, we assessed the independent effect of V‐LH in both study samples by incorporating additional genetic confounders to the applied regression models and adjusted the association test for the three SNPs (*FSHB* ‐211 G/T, rs10835638; *FSHR* ‐29 G/A, rs1394205; *FSHR* c.2039 A/G, rs6166) previously shown to modify reproductive parameters (Grigorova *et al*., 2008, 2013, 2014). Results of association testing in the two study samples were combined in meta‐analysis implemented in the meta package (Schwarzer, 2010, http://CRAN.Rproject.org/package=meta) developed for the statistical package R (www.r-project.org). Meta‐analysis was based on inverse variance method under fixed effects model. The results of meta‐analysis were subjected to Bonferroni correction for multiple testing with an estimated threshold 0.05/14 = 3.6 × 10^−3^, taking into account the number of independent measurements (seven) and sample sets (two).

Statistical differences between the groups of V‐LH carriers (G/G homozygotes and A/G‐heterozygotes) and non‐carriers (A/A‐homozygotes) in hormonal, testicular and semen parameters were assessed by the non‐parametric Mann–Whitney *U*‐test (PASW software Grad Pack 18.0, SPSS Inc., Chicago, IL, USA).

## Results

### No enrichment of V‐LH among male infertility patients

The Baltic young men cohort (*n* = 986, aged: 20.1 ± 2.1 years; Table 1A) and Estonian idiopathic infertility group (*n* = 607, aged: 35.1 ± 5.9 years; Table 1A) were genotyped for a common polymorphism (Trp8Arg; rs1800447, A‐>G) determining the V‐LH isoform. All subjects carrying LHβ Arg8 variant were confirmed to carry also Thr15 allele. The determined genotype frequencies were consistent with HWE in both study samples (*p *>* *0.5). No significant difference was detected between young men and infertility patients for the V‐LH allele frequency (11.0 vs. 9.3%, respectively; Table**** 1B).

### Higher LH among the carriers of V‐LH in both study samples

**Figure 2:**
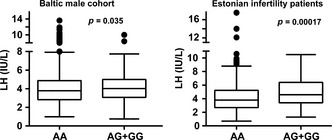
Tukey boxplots for the distribution of serum LH values in the Baltic young men cohort (AA, *n* = 780, AG+GG, *n *= 206) and in the Estonian idiopathic infertility patient sample (AA, *n* = 500, AG+GG, *n* = 107) sub‐grouped according to the carrier status of the V‐LH variant (rs1800447, G‐allele). *p*‐value of Mann–Whitney *U*‐test is shown.

V‐LH was associated with higher serum LH in both, the Baltic young men cohort (linear regression additive model: *p *=* *0.022, allelic effect = 0.26 IU/L) and Estonian idiopathic infertility group (*p *=* *0.008, effect = 0.59 IU/L) (Tables**** 2, 3). In meta‐analysis, the statistical significance was enhanced (*p *=* *0.0007, resistant to Bonferroni correction for multiple testing; effect = 0.33 IU/L; Table 4). The increase in serum LH level among the V‐LH carriers (AG+GG genotypes) compared to the WT‐LH homozygotes (AA‐genotype) in the infertility patient group (5.0 ± 2.3 vs. 4.2 ± 2.0 IU/L; Mann–Whitney *U*‐test, *p *=* *0.00017) was more pronounced compared to the young men cohort (4.2 ± 1.5 vs. 4.0 ± 1.7 IU/L; *p *=* *0.035) (Fig.**** 2).

**Table 2:** Male reproductive parameters in the Baltic young men cohort stratified by the V‐LH (A‐>G) genotype

Parameter^a^	A/A (*n *=* *780)	A/G (*n *=* *196)	G/G (*n *=* *10)	Standard cofactors^b^		Standard cofactors and genetic cofactors^b^^,^ c	
				*p*‐value	G‐allele effect	*p*‐value	G‐allele effect
LH (IU/L)	4.0 ± 1.7 3.8 (2.8–4.9)	4.2 ± 1.5 4.0 (3.1–5.0)	4.5 ± 1.6 4.3 (3.1–5.6)	**0.022**	0.26 (0.11)	**0.022**	0.27 (0.11)
Total testosterone (nmol/L)	27.5 ± 9.2 26.6 (20.8–32.7)	27.2 ± 9.0 26.1 (20.9–32.7)	30.3 ± 7.6 28.3 (25.5–38.2)	0.931	0.06 (0.65)	0.979	0.02 (0.65)
Free testosterone (%)	2.3 ± 0.4 2.3 (2.0–2.6)	2.3 ± 0.4 2.3 (2.0–2.6)	2.2 ± 0.4 2.0 (1.9–2.5)	0.287	−0.03 (0.03)	0.274	−0.03 (0.03)
Estradiol (pmol/L)	92.9 ± 24.5 89.0 (76.0–106.0)	97.9 ± 28.0 94.0 (81.0–110.8)	92.0 ± 29.4 90.0 (67.3–104.0)	0.120	5.37 (3.42)	0.131	5.34 (3.49)
SHBG (nmol/L)	34.4 ± 14.3 32.0 (25.0–41.0)	34.6 ± 12.8 33.0 (25.3–43.0)	38.8 ± 10.4 43.0 (29.5–46.0)	0.314	0.91 (0.90)	0.298	0.95 (0.91)
FSH (IU/L)	3.1 ± 1.7 2.8 (2.0–3.9)	2.9 ± 1.5 2.6 (1.7–3.6)	3.6 ± 1.5 3.7 (2.7–4.4)	0.143	−0.14 (0.10)	0.258	−0.11 (0.10)
Inhibin B (pg/mL)	232.3 ± 78.3 223.0 (177.0–278.0)	215.4 ± 78.5 208.5 (163.0–260.0)	224.1 ± 63.7 214.5 (194.0–268.0)	0.218	−11.00 (9.36)	0.178	−12.24 (9.54)
Total testes volume (mL)	49.4 ± 10.6 50.0 (41.0–56.0)	48.0 ± 9.2 49.5 (41.8–53.0)	43.8 ± 8.2 43.0 (35.8–103.3)	**0.047**	−1.47 (0.76)	**0.011**	−1.88 (0.76)
Total sperm count per ejaculate (× 10^6^)	280.1 ± 275.2 213.3 (105.2–362.8)	236.6 ± 211.8 187.5 (86.9–321.1)	305.7 ± 231.6 316.2 (215.1–423.7)	0.223	−15.69 (14.01)	0.146	−18.84 (14.25)
Sperm concentration (× 10^6^/mL)	82.9 ± 74.9 65.9 (35.2–108.2)	70.8 ± 59.8 58.3 (31.5–96.1)	103.6 ± 49.1 115.8 (52.0–131.9)	0.234	−4.46 (4.04)	0.155	−5.37 (4.11)
Semen volume (mL)	3.5 ± 1.6 3.3 (2.3–4.5)	3.4 ± 1.6 3.2 (2.3–4.5)	3.2 ± 0.9 3.2 (2.5–4.0)	0.784	−0.03 (0.12)	0.744	−0.04 (0.12)
AB motile (%)	57.0 ± 13.5 58.0 (50.0–66.0)	57.6 ± 13.5 59.0 (49.0–67.3)	56.0 ± 15.6 59.0 (47.0–68.0)	0.792	0.31 (1.19)	0.866	0.20 (1.20)

a Data are presented as mean **± **SD and median (25th to 75th percentile).

b Marker trait association testing was performed using linear regression under additive model with the adjustment for age, BMI, smoking status and recruitment centre. Hormone measurements were additionally corrected for blood sampling hour and semen parameters were corrected for ejaculation abstinence period; nominal *p *<* *0.05 has been highlighted in bold.

c Linear regression model was additionally adjusted for *FSHB* rs10835638, *FSHR* rs6166 and rs1394205.

**Table 3:** Male reproductive parameters in the Estonian idiopathic infertility group stratified by the V‐LH (A‐>G) genotype

Parameter^a^	A/A (*n *=* *500)	A/G (*n *= 101)	G/G (*n *= 6)	Standard cofactors^b^		Standard cofactors and genetic cofactors^b^^,^ c	
				*p*‐value	G‐allele effect	*p*‐value	G‐allele effect
LH (IU/L)	4.2 ± 2.0 3.8 (2.7–5.2)	5.1 ± 2.3 4.6 (3.6–6.4)	4.2 ± 3.0 3.2 (1.9–7.2)	**0.008**	0.59 (0.21)	**0.009**	0.60 (0.22)
Total testosterone (nmol/L)	18.6 ± 6.3 18.0 (14.0–22.0)	19.3 ± 6.3 18.7 (14.4–23.8)	16.5 ± 8.4 14.7 (9.1–24.3)	0.304	0.88 (0.85)	0.280	0.93 (0.86)
Estradiol (pmol/L)	101.1 ± 37.2 89.8 (73.4–116.0)	101.0 ± 41.5 84.5 (73.4–117.5)	106.8 ± 53.3 76.4 (73.0–156.0)	0.890	−0.33 (2.41)	0.915	−0.26 (2.43)
FSH (IU/L)	7.3 ± 6.0 5.6 (3.5–8.8)	6.2 ± 5.5 5.0 (3.4–7.0)	6.7 ± 5.8 4.2 (2.1–13.6)	**0.018**	−0.85 (0.41)	**0.019**	−0.84 (0.40)
Total testes volume (mL)	40.3 ± 10.2 40.0 (34.0–46.0)	39.7 ± 9.7 40.0 (33.5–47.0)	47.0 ± 9.9 47.0 (39.5–55.5)	0.635	−0.52 (1.11)	0.563	−0.64 (1.14)
Total sperm count per ejaculate (× 10^6^)	35.0 ± 31.2 27.0 (9.2–52.6)	34.3 ± 31.6 25.6 (9.5–53.4)	24.4 ± 21.9 14.6 (7.2–49.9)	0.331	−2.18 (2.56)	0.303	−2.33 (2.59)
Sperm concentration (10^6^/mL)	8.1 ± 5.9 7.2 (2.6–13.0)	8.1 ± 5.9 7.0 (2.6–13.0)	5.1 ± 4.2 3.9 (1.5–9.2)	0.557	−0.29 (0.53)	0.503	−0.33 (0.54)
Semen volume (mL)	4.3 ± 1.8 3.9 (3.0–5.3)	4.1 ± 1.7 4.0 (3.0–5.3)	4.6 ± 1.3 4.2 (3.7–5.7)	0.531	−0.11 (0.19)	0.597	−0.10 (0.19)

a Data are presented as mean ± SD and median (25th to 75th percentile).

b Marker trait association testing was performed using linear regression under additive model with the adjustment for age; semen parameters were additionally corrected for the ejaculation abstinence period; nominal *p *<* *0.05 has been highlighted in bold.

c Linear regression model was additionally adjusted for *FSHB* rs10835638, *FSHR* rs6166 and rs1394205.

**Table 4:** Results of meta‐analysis across the Baltic male cohort and Estonian infertility group

Parameter	Standard cofactors		Standard cofactors and genetic cofactors	
	*P*‐value	G‐allele effect	*p*‐value	G‐allele effect
LH (IU/L)	**0.0007** a	0.33 (0.10)	**0.0006** a	0.34 (0.10)
Total testosterone (nmol/L)	0.483	0.36 (0.52)	0.499	0.35 (0.52)
Estradiol (pmol/L)	0.428	1.56 (1.97)	0.432	1.57 (1.99)
FSH (IU/L)	0.064	−0.18 (0.10)	0.115	−0.15 (0.10)
Total testes volume (mL)	0.063	−1.17 (0.63)	**0.018**	−1.50 (0.63)
Total sperm count per ejaculate (× 10^6^)	0.299	−2.62 (2.52)	0.262	−2.86 (2.55)
Sperm concentration (10^6^/mL)	0.493	−0.36 (0.53)	0.438	−0.42 (0.54)
Semen volume (mL)	0.603	−0.05 (0.10)	0.574	−0.06 (0.10)

Nominal *p *<* *0.05 has been highlighted in bold.

a Significant after Bonferroni correction for multiple testing; α = 0.05/14 = 3.6 × 10^−3^ (7 independent measurements in two samples).

In the Baltic male cohort, a suggestive association was detected between V‐LH and lower total testes volume (linear regression additive model: *p *=* *0.047; allelic effect −1.47 mL; Table 2). Decreased serum FSH was observed among the V‐LH carriers in the Estonian infertility patient group (*p *=* *0.018; effect = −0.85 IU/L; Table 3). However, the meta‐analysis across the two study samples did not provide conclusive support for these pilot observations. Serum total testosterone, estradiol, SHBG and sperm parameters were not associated with V‐LH carrier status either in the separate analysis of the two study groups or in the combined meta‐analysis.

### Effect of V‐LH on LH and total testes volume independent of other known genetic risk factors

To demonstrate the independent genetic effect of V‐LH, linear regression analysis was performed with additional adjustm‐ent for the genotypes of the three SNPs (rs10835638, *FSHB* ‐211, G/T; rs6166, *FSHR*, Asn680Ser; rs1394205, *FSHR* ‐29, G/A) known to modify the levels of serum LH. The statistical significance (resistant to Bonferroni correction) and the effect of V‐LH on serum LH level remained unchanged in the separate analysis of both study samples, as well as in the meta‐analysis combining the obtained results (Tables 2, 3, 4).

## Discussion

In this study, a comprehensive set of hormonal, testicular and seminal parameters was analysed to elucidate the effect of V‐LH in two large and well‐characterized andrological samples – the Baltic cohort of young men and the Estonian male idiopathic infertility patient group. The carrier frequency of V‐LH (heterozygotes and homozygotes) in the two study groups (20.9 and 17.6%, respectively. Table**** 1B) was concordant with the previous report on V‐LH frequency among Estonians determined by the immunoassay‐based isoform detection method (21.3%; Nilsson *et al*., 1997). There was no difference in allele and genotype frequencies of V‐LH between idiopathic infertility patients and a population‐based cohort of young men, confirming previous studies (Ramanujam *et al*., 2000; Lee *et al*., 2003). As the major result, we detected a convincing association between the V‐LH carrier status and increased serum levels of LH in both study groups. Meta‐analysis conclusively supported this evidence as the statistical significance was enhanced and reached a *P*‐value resistant to Bonferroni correction for multiple testing (Tables 2, 3, 4; Fig. 2). We further demonstrated an independent effect of V‐LH on circulating LH concentration by applying an additional adjustment of the analysis for the SNPs previously demonstrated to affect LH levels (Grigorova *et al*., 2008, 2013, 2014).

There are several lines of published evidence supporting our study outcome. Considering that the profile of oligosaccharide side chains modulates the half‐life of pituitary gonadotropins, there is a reason to believe that the Trp8Arg/Ile15Thr substitutions modify the effect of LH in vivo (Morell *et al*., 1971; Strott, 2002). LH molecules with lower number of sulfonated and higher amount of sialic acid residues were reported to survive longer in the human circulation (Wide *et al*., 2009). Compared to WT‐LH, V‐LH has higher number of sialic acid residues and indeed, its measured half‐life is approximately 40% longer (half‐life: WT‐LH 108 min, V‐LH 148 min; Wide *et al*., 2010). The V‐LHβ polymorphism was suggested to have originated through an ancient gene conversion event, where one of the hCGβ encoding *CGB* genes has acted as a conversion donor and the *LHB* gene as an acceptor of the respective genomic fragment (Hallast *et al*., 2005). The concerted substitutions in V‐LH exon 2 leading to Trp8Arg/Ile15Thr correspond to the nucleotide sequence in the respective positions of all six *CGB* genes (Nagirnaja *et al*., 2010). Because of the Trp8Arg/Ile15Thr substitutions in the encoded LHβ subunit it resembles the more stable hCGβ and subsequently, the V‐LH has a prolonged half‐life compared to WT‐LH. Additionally, V‐LHβ has been associated with a promoter differing from WT‐LHβ in eight nucleotide positions and exhibiting ~40% higher basal transcriptional activity (Jiang *et al*., 1999). In concordance with these data, we report significantly higher serum level of LH in V‐LH carriers among the young Baltic men and Estonian male infertility patients. Consistently, young female V‐LH carriers were also measured increased LH (*n* = 120, age: 31.4 ± 3.6 years; Du *et al*., 2012).

In contrast, in a cohort of middle‐aged and elderly men (*n* = 2748; mean age: 60.2 ± 11.2 year) significantly lower serum LH levels among V‐LH carriers compared to the WT‐LH homozygotes were reported (Huhtaniemi *et al*., 2010). During ageing, the changes in hormonal pattern gradually lead to primary testicular dysfunction with declining testosterone levels and an associated elevation of LH (Araujo & Wittert, 2011). We speculate that despite the higher basal activity, the V‐LHβ promoter is unable to respond to the increased physiological requirement to the similar degree as the WT‐LH promoter. In ageing men, the WT‐LH homozygotes have increasing levels of serum LH, which is not the case for the V‐LH carriers.

Our observation that V‐LH shows a trend for association with lower testes volume among the young Baltic men is in concordance with the few conducted clinical studies showing lower in vivo potency of V‐LH compared to WT‐LH. The association between V‐LH and reduced testes volume has been previously reported in a small study (49 boys) on pubertal onset and progression, where V‐LH carriers additionally exhibited slower pubertal progression and growth rate (Raivio *et al*., 1996). Another study reported increased prevalence of V‐LH among cryptorchid boys born with gestational age > 40 weeks and suggested that the lower hormonal efficacy of V‐LH predisposes for improper testicular descent in late pregnancy (Kaleva *et al*., 2005). However, as the effect in total testes volume was found only in the young male cohort, it needs to be confirmed in further studies. The same applies to the pilot observation of lower FSH among the V‐LH carriers in the Estonian infertility patient group, which may also represent a chance finding as a result of a severely altered reproductive physiology in these patients.

In summary, this study demonstrates for the first time a conclusive contribution of V‐LH to the natural variance in male serum LH levels. Its downstream clinical consequences are still to be learned.

## Author Contributions

ML, MP, MG and AMP conceived and designed the experiments. AMP, MA and TK performed the experiments. AMP, MG and ML analysed the data. ML and MP contributed reagents/materials/analysis tools. AMP, ML and MP wrote the manuscript. MP, OP, VV, BŽ, JE and VM contributed to recruitment and clinical phenotyping of patients. MG, MA, OP, VV, BŽ, JE, VM and TK were involved in critical commenting of the data and manuscript.

## Conflict of Interest

None.

## Supporting information


**Table S1.** Potential confounders incorporated in genetic tests: age, BMI, ejaculation, blood sampling hour.
