# Supplementary material for: ‘Carriers of V‐LH among 1593 Baltic men have significantly higher serum LH’
Source: Andrology. 2015 Mar 26;3(3):512–9. doi: 10.1111/andr.12022 (PMC4832392; doi:10.1111/andr.12022)
Supplement: Supplementary file 1 — Table S1. Potential confounders incorporated in genetic tests: age, BMI, ejaculation, blood sampling hour. [file ANDR-3-512-s001.docx]

**Supporting information**

Supporting Table 1. Potential confounders incorporated in genetic tests: age, BMI, ejaculation, blood sampling hour.

| Parameter | A/A  (n=780) | A/G  (n=196) | G/G  (n=10) |
| --- | --- | --- | --- |
| Age | 20.2±2.0  19.8 (18.5-21.5) | 20.1±2.0  19.8 (18.5-21.3) | 18.8±1.6  18.8 (17.6-19.3) |
| BMI | 22.4±2.6  22.1 (20.7-23.7) | 22.4±2.6  22.2 (20.9-23.5) | 21.7±1.8  21.8 (20.1-23.2) |
| Ejaculation abstinence period (hours) | 107.7±63.6  86 (63-134) | 106.0±61.7  86 (62-135.3) | 126.5±52.2  130 (80-156.5) |
| Blood sampling hour (minutes after 8am) | 147.5±76.4  125 (100-180) | 147.8±72.9  140 (100-180) | 161.0±93.1  170 (66.3-215) |

Data are presented as mean ± SD and median (25-75^th^ percentile)
